# On the Action of Light in Small-Pox

**Published:** 1871-05

**Authors:** J. H. Waters


*On the Action of Light in Small-Pox.* By J. H. Waters, M.B., M.C., &c.

[From the London Lancet, Feb. 4.]

It has long been known that chemical changes can be effected by light, both of combination and of decomposition, especially the latter, and that substances in great numbers are found more or less susceptible to its influence. Many instances might be given of this. Chlorine and hydrogen at ordinary temperatures will not combine except when acted upon by light. In organic chemistry examples in plenty abound, as the salts of silver, &c. It is generally the luminous parts of a ray of light that effect this. In them lie the chief chemical action, and this exists even when the light is feeble, although no doubt for every kind of ray a substance might be found which, if circumstances were favorable, would be acted upon by it. This power has received the name of actinism, and is distinguishable from the heat-giving rays by being refrangible to the highest degree.

Animal and vegetable nature are peculiarly susceptible to the influence of light, their color being in a great measure dependent upon its action. If a plant is allowed to grow for any length of time in the dark, the leaves and stalks are destitute of color, and it bears no seed. An animal living in the dark loses its color. Man especially is most sensitive to light, his skin becoming of a peculiar waxy hue when he is deprived of it; and when constantly exposed to its effects his face and hands get deeply colored. This is in persons with their ordinary health.

The sensibility of the skin varies greatly in different parts of the body. Weber, to prove this, applied the points of a pair of compasses to it. In some places the two points can be felt when only a few lines apart; in others they must be separated to a considerable extent to be distinguished; and it is those parts exposed constantly to the action of light that are most sensitive. This would show some connection between nerve development and light, which as yet is not understood. Disease modifies this sensibility considerably by its action. The skin may be rendered more highly sensitive, or less so; or sensation may be altered and perverted. This, although well known to be due to some change in the nervous system, is far from being understood.

Those diseases that attack the skin, or rather that are eliminated by it, and which increase its susceptibility to light, are more dangerous when exposed to its influence. Small-pox, in particular, is more severe when light is allowed thoroughly to pervade the patient's sick room. John of Gaddesden first noticed this, and proposed its exclusion from those chambers where patients suffering from this disease lay; since his time many physicians have acted upon his idea, with more or less success as they have efficiently carried it out. If white light is entirely excluded from the patient, there is no doubt the disease is less severe; by white light I mean daylight. The room being so darkened that not even a single ray can enter it, and a candle being used instead, the effect is to arrest the disease at the papular or vesicular stage; it never becomes purulent, and the skin between the vesicles is never inflamed or swollen; the liquor sanguinis is prevented from becoming pus; we never see the large scabs of matter covering the face; there is no intense pain; even the itching is trifling; and the smell, so annoying to all concerned, is, if not altogether removed, so diminished as to be easily borne. The earlier in the disease the room is darkened, the more certain will the effects I have named follow; but if, during the stages of primary fever or eruption, the white rays of light are admitted only for a short time, it is sufficient to cause great mischief, and to nullify to an immense extent all that has been before done.

Another advantage derived from this mode of treatment is that we are able to administer medicines which it would be impossible to use without it—those that act upon the skin and assist it to eliminate its poisons; the inflammatory action being less severe, we run no danger by stimulating its excretory powers in moderation.

The treatment I follow, in addition to the darkening of the room and quiet in bed, is to give the patient a farinaceous diet, with beef-tea and fish, ripe fruits and milk, lemonade, soda-water, barley-water, and demulcent drinks. The room to be well ventilated; this can be effected by the window remaining open under the covering that excludes the light. Tepid sponging, with frequent changes of linen. Purgatives should be given very cautiously; none but the mildest are admissible, and it is better to regulate the bowels with fruits than to use any at all; if a laxative is necessary, mild enemata are the best. From the commencement of the fever to the acumination of the pock, arsenic (Fowler's solution) with iodine in small doses, the iodide of potassium, and solution of the acetate of ammonia, in a mixture, a dose every four to six hours, is in most cases well borne and does good; after this, arsenic (a solution of the arsenite of soda) with the syrup of the phosphate of iron will act better, and be a restorative tonic. Stimulants may be given if necessary; and if sleeplessness is complained of, chloral hydrate, with or without opium or henbane, is best.

I find, by reference to my notes of a great number of cases which have been under this treatment, that when the patient has been seen early—that is, before the appearance of the eruption—the general history is as follows:

First, second, and third days.—Patient is suffering from fever, and from the well-known symptoms of small-pox. Has been complaining some days. The room to be darkened; tepid sponging, &c., with the first arsenical mixture to be given; quiet in bed enjoined; milk diet, &c.

Fourth day.—Eruption is beginning to show itself; less fever, &c., but itching of the skin has commenced. No powder to be used, as this I find does more harm than good; it fills up the pores of the skin, preventing perspiration, either insensible or otherwise. Cold cream, the lime liniment, or sponging, to be used instead. The same mixture, &c., to be repeated.

Fifth day.—The eruption well out, with slightly inflamed base; less fever. The same treatment continued.

Sixth, seventh, and eighth days of the disease (third, fourth, and fifth of the eruption).—The eruption progresses in the ordinary manner, but the fever has nearly subsided, except in the more severe forms of the disease. Appetite returning. Same treatment continued.

Eighth, ninth, and tenth days of the disease (sixth, seventh, and eighth of the eruption).—The vesicles, instead of being converted into pustules, get less and less, their contents being absorbed, or they dry up into a brown scab, which comes away in the ordinary manner. There is no secondary fever. The patient feels quite well, eats well, and is in fact free from the disease.

This is the ordinary progress of the disease under the treatment I have mentioned; the exceptions are such as only prove the rule.

I was attending four children suffering from small-pox. They were so well on the fifth day of the eruption that they were allowed to play about the room. The nurse having to go away for a short time, they got to a window, and pulled down a carpet which had been used to exclude the light. The window being open for ventilation, the two eldest put their heads out, and thus nullified the precautions before taken. When I saw them, a few hours afterwards, they were feverish, and the eruption looked very angry. These two children got well, but were marked; the other two were not.

A lady, who was staying with some friends, having been obliged to quit Paris just before the siege of that city, brought the small-pox with her. The eruption was coming out when I first saw her. Went on well till the fourth day—so well that she wished to read. To admit sufficient light for the purpose, the nurse pulled up the corner of the green baize. After the lapse of a few hours, tingling heat and pain, with fever, came on; and, this being a confluent case, she became very ill; the secondary fever was very severe. She recovered, but was marked.

The son of a shoemaker was attacked by the disease. I saw him on the first day of the eruption. The necessary precautions for darkening the room were not carried out properly in my absence. When I was away, light was admitted. This was a confluent case, also, in the face. He never did well, and died on the eleventh day. The other three children all had it. All did well, and were not marked. The treatment was followed out with them strictly.

